# Supplementary material for: The genome sequence of the Manchester Treble-Bar, Carsia sororiata (Hubner, 1813) (Lepidoptera: Geometridae)
Source: Wellcome Open Res. 2026 Sep 28;11:772. [Version 1] doi: 10.12688/wellcomeopenres.27969.1 (PMC13760148; doi:10.12688/wellcomeopenres.27969.1)
Supplement: Peer review history

# Reviewer report: Reviewer response for version 1

**DOI:** 10.21956/wellcomeopenres.30771.r178340

**Published:** 2026-11-12

**Authors:** Mohadeseh Tahami

**Affiliations:** 1 University of Jyväskylä, Jyväskylä, Finland

The study presents the whole genome sequence and chromosome assembly of Manchester Treble-Bar, *Carsia sororiata,* belonging to Geometridae moth species. Morphological characteristics of the species along with its habitat distribution and the species life cycle are presented. 

 Identification was done both morphologically and via DNA barcoding.

 The study used PacBio and Hi-C techniques from an adult male to generate sequence data suitable for scaffolding and chromosome profiling. PacBio sequencing yielded ~4 million raw reads with140x coverage. The genome was assembled into 2 haplotypes, and the first haplotype was used for chromosome scaffolding with the total length of 317.73 Mb leading to BUSCO completeness of 98%.

 The authors thoroughly explain the species background, the rationale for the genome sequencing and the NGS techniques. More details on the bioinformatics part could help the data reproducibility for example, the pipeline versions and if default parameters were used for each step or otherwise.

Are the datasets clearly presented in a usable and accessible format, and the assembly and annotation available in an appropriate subject-specific repository?

Yes

Are sufficient details of the sequencing and extraction, software used, and materials provided to allow replication by others?

Partly

Are the rationale for sequencing the genome and the species significance clearly described?

Yes

Are the protocols appropriate and is the work technically sound?

Yes

Reviewer Expertise:

taxonomy, NGS, phylogeny

I confirm that I have read this submission and believe that I have an appropriate level of expertise to confirm that it is of an acceptable scientific standard.

# Reviewer report: Reviewer response for version 1

**Published:** 2026-09-28

**Authors:** Automated Benchmarking

**Affiliations:** 1 Wellcome Sanger Institute, Hinxton, UK

As this article is a Genome Note, Automated Benchmarking has been used to determine whether the reported assembly passes four defined checks established by the Earth BioGenome Project: the raw and assembled data are publicly available, the Earth BioGenome Project three-part metric is met, the k-mer completeness exceeds 90%, and the BUSCO score passes the stated threshold. For Carsia sororiata (ilCarSoro1), all four checks passed. Data available: pass. ENA study PRJEB82165, raw-read accessions ERR13946460 and ERR13947479, and assembly accessions GCA_964340635.1 and GCA_964340605.1 are public. Earth BioGenome Project three-part metric: pass. Haplotype 1 is reported as 6.C.Q63, meeting the 6.C.Q40 reference standard. k-mer completeness: pass. The combined haplotype assemblies have 99.77% k-mer completeness, exceeding the Earth BioGenome Project target of 90%. BUSCO: pass. Haplotype 1, which is proposed as the reference assembly for this species, has 98.4% complete BUSCOs (97.4% single-copy and 0.9% duplicated). BUSCO software version: 5.5.0; lineage dataset: lepidoptera_odb10. It meets the target of greater than 90% complete and single-copy BUSCOs. This decision uses values reported in the Genome Note and public accession records.

Reviewer Expertise:

NA

I confirm that I have read this submission and believe that I have an appropriate level of expertise to confirm that it is of an acceptable scientific standard.
